# Supplementary material for: A genome-scale single-cell CRISPRi map of trans gene regulation across human pluripotent stem cell lines
Source: Cell Genom. 2025 Dec 1;6(2):101076. doi: 10.1016/j.xgen.2025.101076 (PMC12903452; doi:10.1016/j.xgen.2025.101076)
Supplement: Document S1. Figures S1–S9, Table S10, and supplemental note [file mmc1.pdf]

Cell Genomics, Volume *6*

Supplemental information

A genome-scale single-cell CRISPRi map of *trans* gene regulation across human pluripotent stem cell lines

Claudia Feng, Elin Madli Peets, Yan Zhou, Luca Crepaldi, Sunay Usluer, Alistair Dunham, Jana M. Braunger, Jing Su, Magdalena E. Strauss, Daniele Muraro, Kimberly Ai Xian Cheam, Marc Jan Bonder, Edgar Garriga Nogales, Sarah Cooper, Andrew Bassett, Steven Leonard, Yong Gu, Bo Fussing, David Burke, Leopold Parts, Oliver Stegle, and Britta Velten

Cell Genomics, Volume *6*

Supplemental information

A genome-scale single-cell CRISPRi map of *trans* gene regulation across human pluripotent stem cell lines

Claudia Feng, Elin Madli Peets, Yan Zhou, Luca Crepaldi, Sunay Usluer, Alistair Dunham, Jana M. Braunger, Jing Su, Magdalena E. Strauss, Daniele Muraro, Kimberly Ai Xian Cheam, Marc Jan Bonder, Edgar Garriga Nogales, Sarah Cooper, Andrew Bassett, Steven Leonard, Yong Gu, Bo Fussing, David Burke, Leopold Parts, Oliver Stegle, and Britta Velten

Supplementary information

A genome-scale single-cell CRISPRi map of *trans* gene regulation across human pluripotent stem cell lines

Claudia Feng[1], Elin Madli Peets[1,*], Yan Zhou[1,*], Luca Crepaldi[1], Sunay Usluer[1], Alistair Dunham[1], Jana M Braunger[6], Jing Su[1], Magdalena E Strauss[1,2], Daniele Muraro[1], Kimberly Ai Xian Cheam[1], Marc Jan Bonder[3], Edgar Garriga Nogales[1], Sarah Cooper[1], Andrew Bassett[1], Steven Leonard[1], Yong Gu[1], Bo Fussing[1], David Burke[4], Leopold Parts[1,@], Oliver Stegle[1,2,3,5@], Britta Velten[1,6,@,#]

[1] Wellcome Sanger Institute, Wellcome Genome Campus, Hinxton, UK
[2] European Bioinformatics Institute, European Molecular Biology Laboratory, Hinxton, UK
[3] Deutsches Krebsforschungszentrum, Heidelberg, Germany
[4] King's College London, London, UK
[5] European Molecular Biology Laboratory, Heidelberg, Germany
[6] Heidelberg University, Heidelberg, Germany

* Contributed equally
# Lead contact
[@] Corresponding author: leopold.parts@sanger.ac.uk (LP), oliver.stegle@embl.de (OS), britta.velten@cos.uni-heidelberg.de (BV)

Supplementary figures

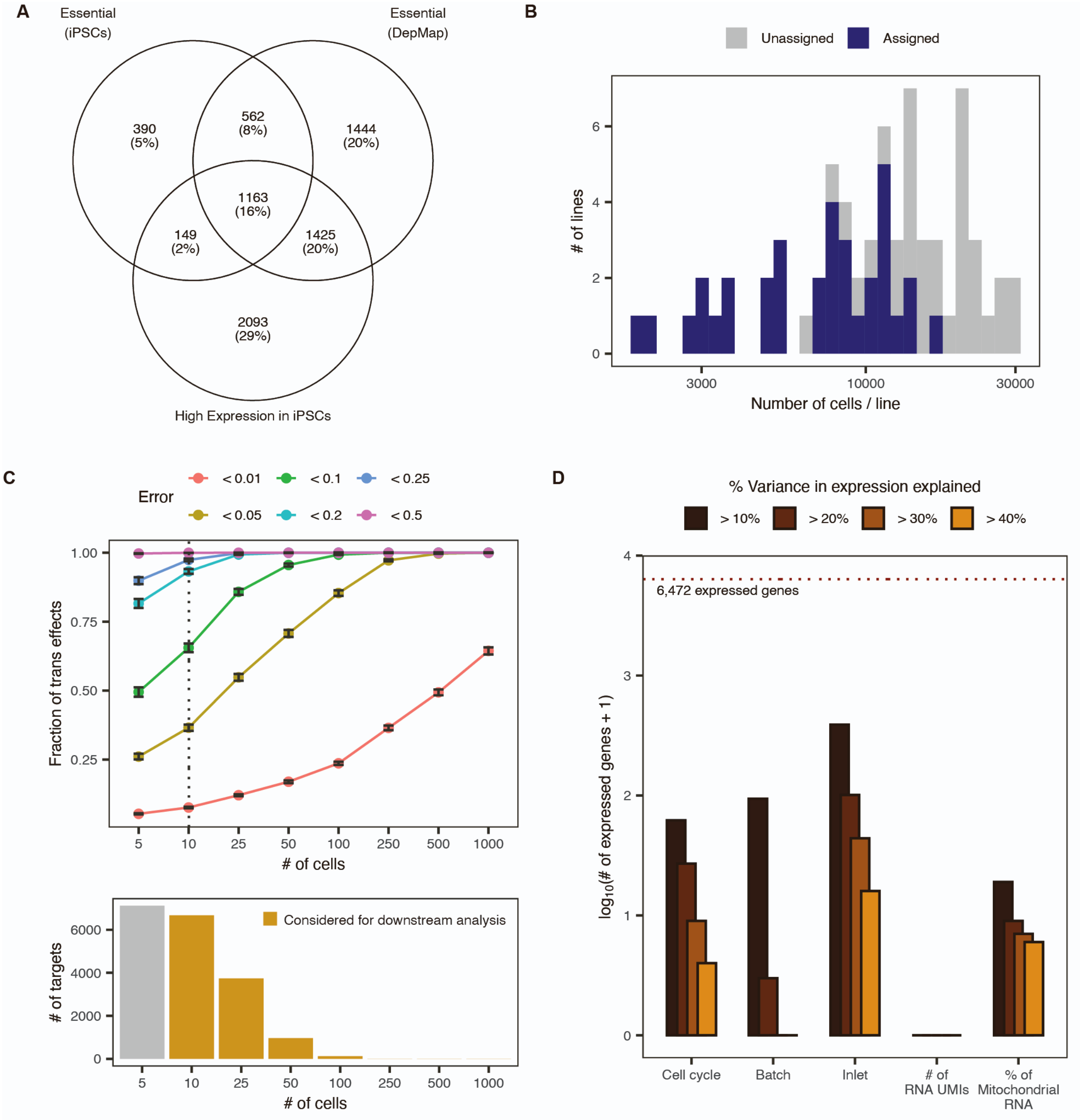

Figure S1 | Coverage and variance in the genome-scale screen, related to Figure 1. A) Venn diagram of genes selected as targets. B) Number of lines (y-axis) for different numbers of cells recovered per line after genotyping (x-axis) with gRNA assigned (blue) and not (grey). C) Estimated absolute error of expression log-fold changes for varying number of assigned cells (top) (relative to estimates from all genes, with a minimum of 1,000; Methods), histogram of cells per target gene (bottom). D) Number of genes (y-axis; log10 scale) with increasing amounts of variance explained (colors) by cell cycle and technical artefacts (x-axis).

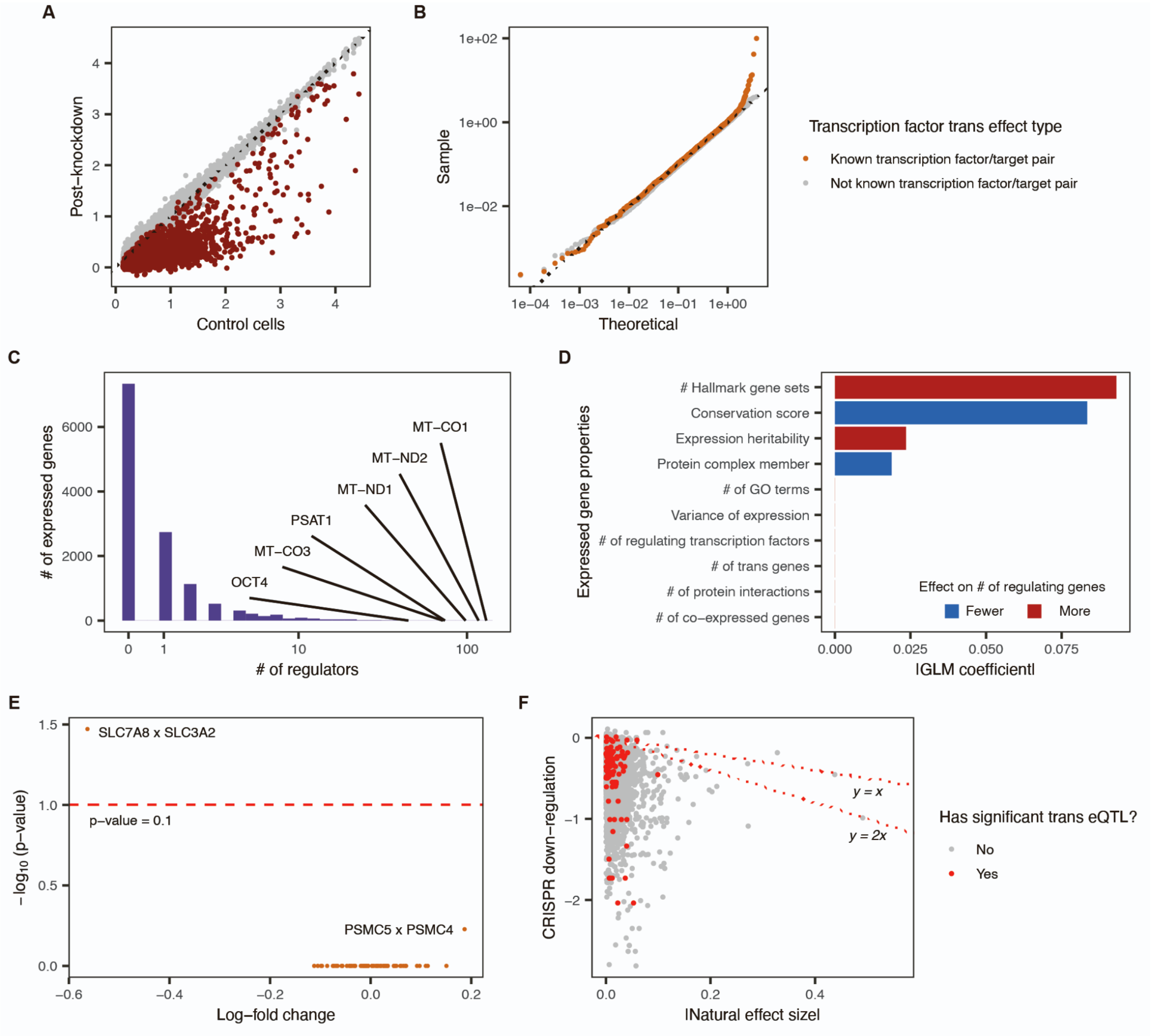

Figure S2 | Molecular signatures of the *trans* effects of gene knockdown, related to Figure 1. A) Down-regulation of target genes due to CRISPRi. Expression of targeted (red markers) and other genes (grey markers) Red dots show target gene expression values in control cells (x-axis) and assigned cells (y-axis), grey dots show expression values of expressed genes beyond the target. B) Quantile-quantile plot of *p*-values of *trans* effect of transcription factors in the DoRoTHeA database. Orange points indicate the *trans* effects of transcription factors and their known targets while gray points indicate *trans* effects the same transcription factors with non-targets. Number of downstream genes (y-axis) with different numbers of regulators (x-axis). Labels: six genes with most upstream regulators. C) Histogram of the number of regulators per expressed gene. Expressed genes with the highest numbers of regulators are labelled. D) Absolute model coefficients (x-axis) for predicting the number of regulators based on properties of the expressed gene (y-axis). Blue: negative coefficients (fewer regulators); red: positive coefficients (more regulators). E) Volcano plot of the log-fold change (x-axis) and log-scale significances (Benjamini Hochberg adjusted p-values, y-axis) for *trans* effects of (target, expressed gene) pairs with a known eQTLs acting in *cis* on the target and *trans* on the expressed gene. Dashed line: p=0.1. Labels: two pairs with corrected p-value less than 1. F) Comparison of effect size of natural variation in expression attributed to a cis eQTLs and CRISPRi. Red: cis eQTLs with at least one significant *trans* effect. Gray: cis eQTLs without any significant *trans* effects.

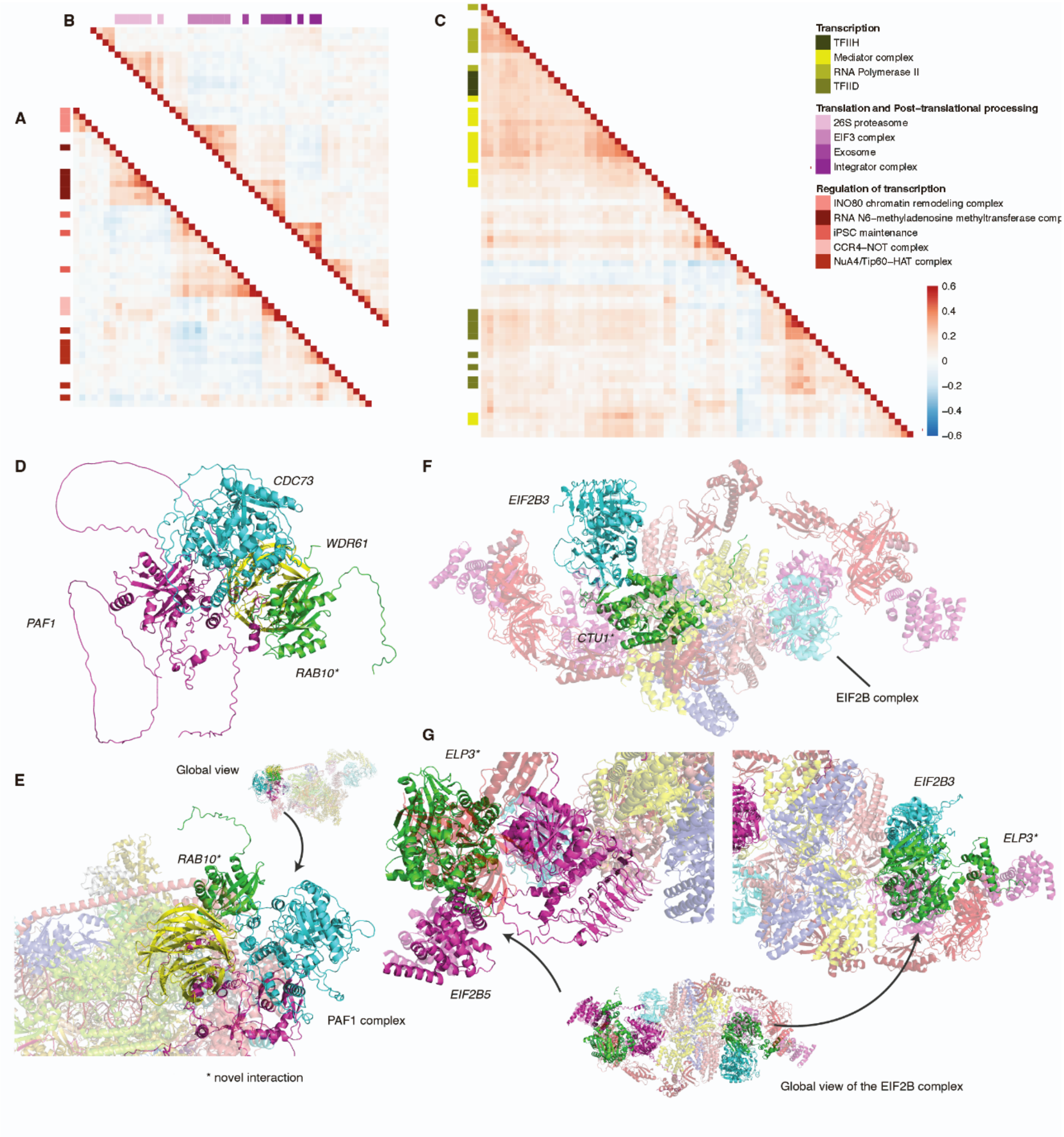

Figure S3 | Plausible protein interactions predicted from similar trans effects of knockdowns, related to Figure 2. Similarity of trans effects between target genes (x- and y-axis) involved in the A) regulation of transcription, B) translation and post-translational processing and C) transcription. Heatmap color: Pearson's R. Annotation color: covariates of biological processes involved (see legend). D) A plausible quadramer formed between *RAB10*, *WDR61*, *CDC73* and *PAF1*. *RAB10* clashes with *CTR9* when we consider the larger structure of Paf[103]. E) Predicted binding structure between *RAB10* and the Paf complex. F) Predicted binding structure of *CTU1* with *EIF2B3* at a buried interface[104] (6O81) of the EIF2B complex. G) Predicted binding structure of *ELP3* with *EIF2B3* and *EIF2B5* of the EIF2B complex.

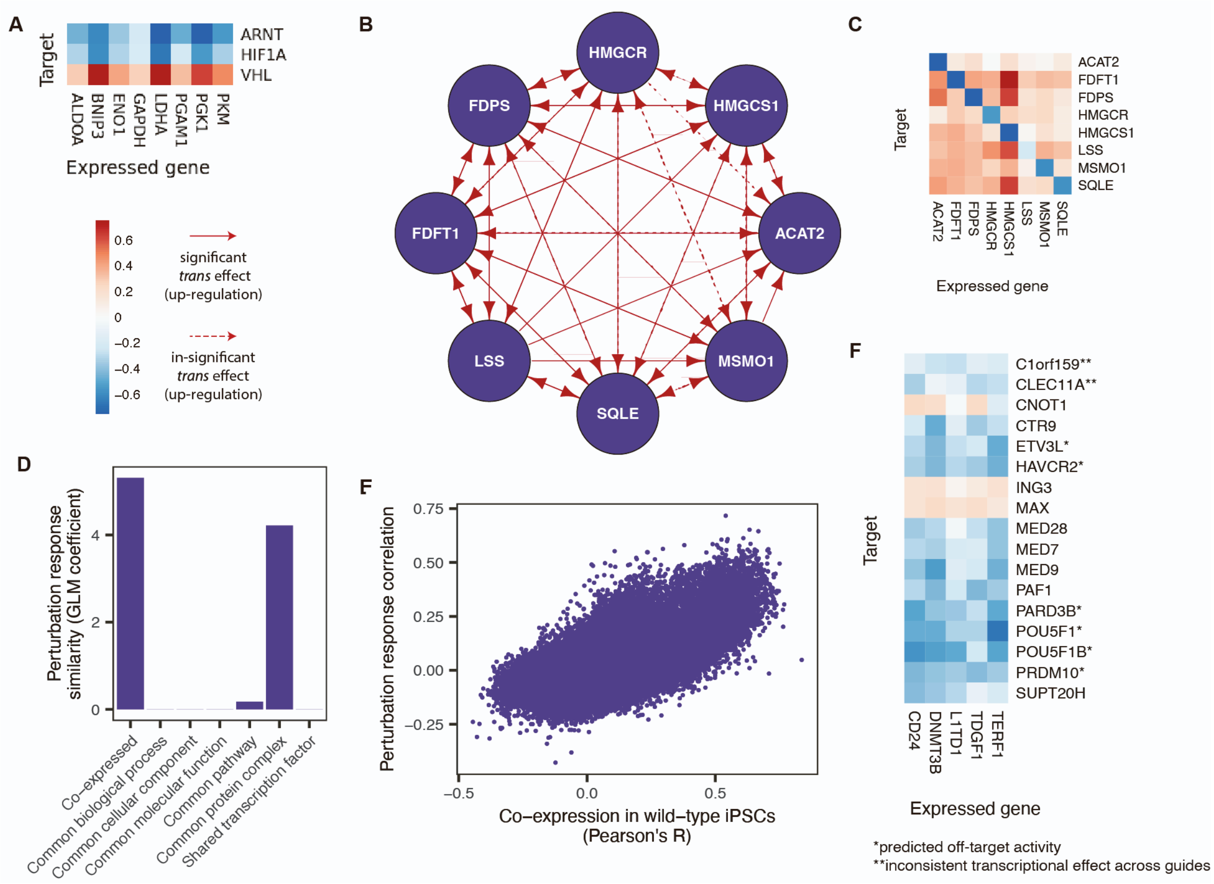

Figure S4 | Co-regulated modules identified by perturbation response similarity, related to Figure 2. A) Heatmap of *trans* effects (log-fold change, color) of genes in the glycolysis pathway (x-axis) due to knockdown of hypoxia pathway regulators *ARNT*, *HIF1A* and *VHL* (y-axis). B) Joint up-regulation of cholesterol biosynthesis pathway members due to down-regulation of a pathway member. Purple nodes: Genes in the cholesterol biosynthesis pathway Red edges: up-regulation of arrow target upon knockdown of arrow source. C) As A), but change (color) of cholesterol biosynthesis gene expression (x-axis) upon knockdown of genes in cholesterol biosynthesis gene expression (y-axis). D) Predicting correlation between co-perturbation profiles of downstream effects. Coefficient (y-axis) for different covariates (x-axis) in a generalized linear model trained to predict correlation of downstream gene log-fold change vectors for pairs of targets. E) Correlation of gene expression values across single cells in wild-type iPSCs (*x*-axis) against correlation in response to perturbations of different targets in CRISPRi screening (*y*-axis). F) As A) and C) but of *trans* effects on iPSC marker genes *CD24*, *DNMT3B*, *L1TD1*, *TDGF1* and *TERF1* (x-axis) due to different target genes (y-axis). Single star: predicted off-target activity on *OCT4* (**Methods**). Double star: inconsistent transcriptional change between guides for the same gene (maximum correlation between guides < 0.1).

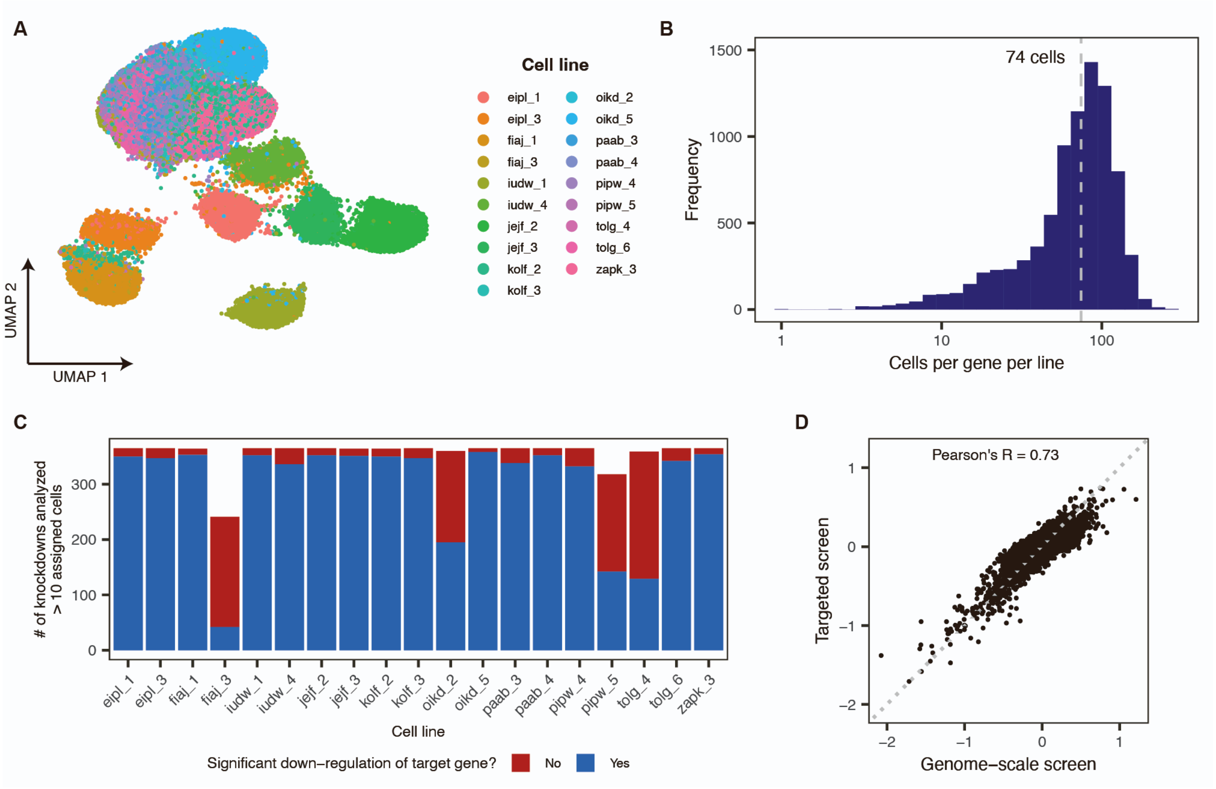

Figure S5 | Recovery of the targeted screen, related to Figure 3. A) UMAP representation (x- and y-axis) of technical covariate corrected expression of all assigned cells (markers) in the targeted screen, Colors: cell lines. B) Number of assigned cells (y-axis) per knockdown per line (x-axis). Dashed line: median number of cells per knockdown per line. C) Number of knockdowns with at least 10 assigned cells, plotted per line. Blue: number of knockdowns with significant (Benjamini-Hochberg adjusted p-value < 0.1, t-test) on-target down-regulation in a line. Red: additional number of knockdowns with insignificant on-target down-regulation. D) Concordance of all *trans* effects that were significant in either the genome-scale or target screen. Log-fold change across all cells in the targeted screen (y-axis) compared to genome-scale screen (x-axis) for 288,089 (target, downstream gene) pairs. A point represents a target-expressed gene pair.

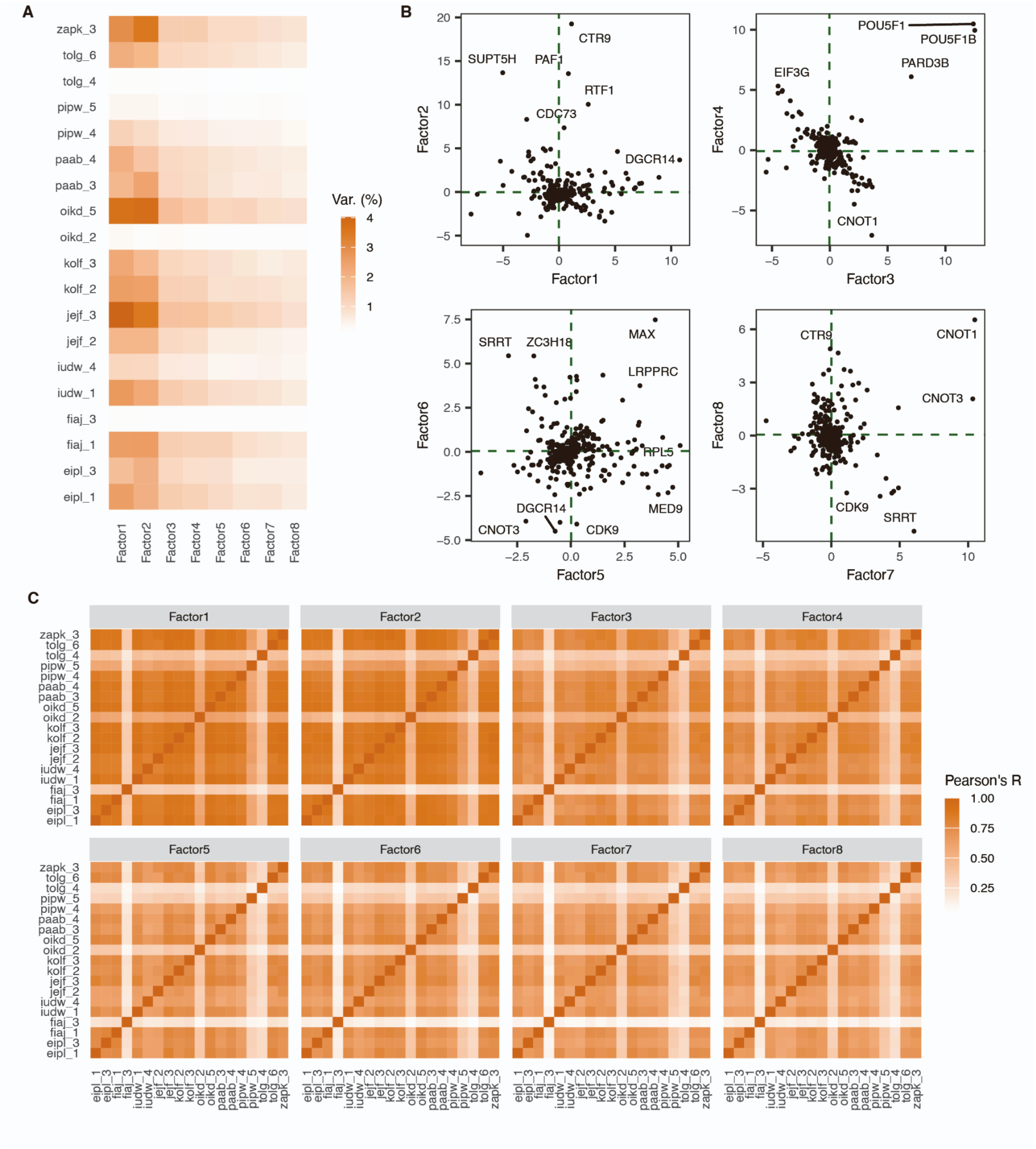

Figure S6 | Global effect of transcriptional change due CRISPRi perturbation across cell lines, related to Figure 3. A) Percentage of log-fold change variance explained (color) by different MOFA factors (x-axis) in different cell lines (y-axis). B) MOFA weights of a knockdown (markers) for different factors (x- and y- axis), for different factor combinations (panels). C) Correlation (color) of MOFA factors (panels), between cell lines (x- and y-axis).

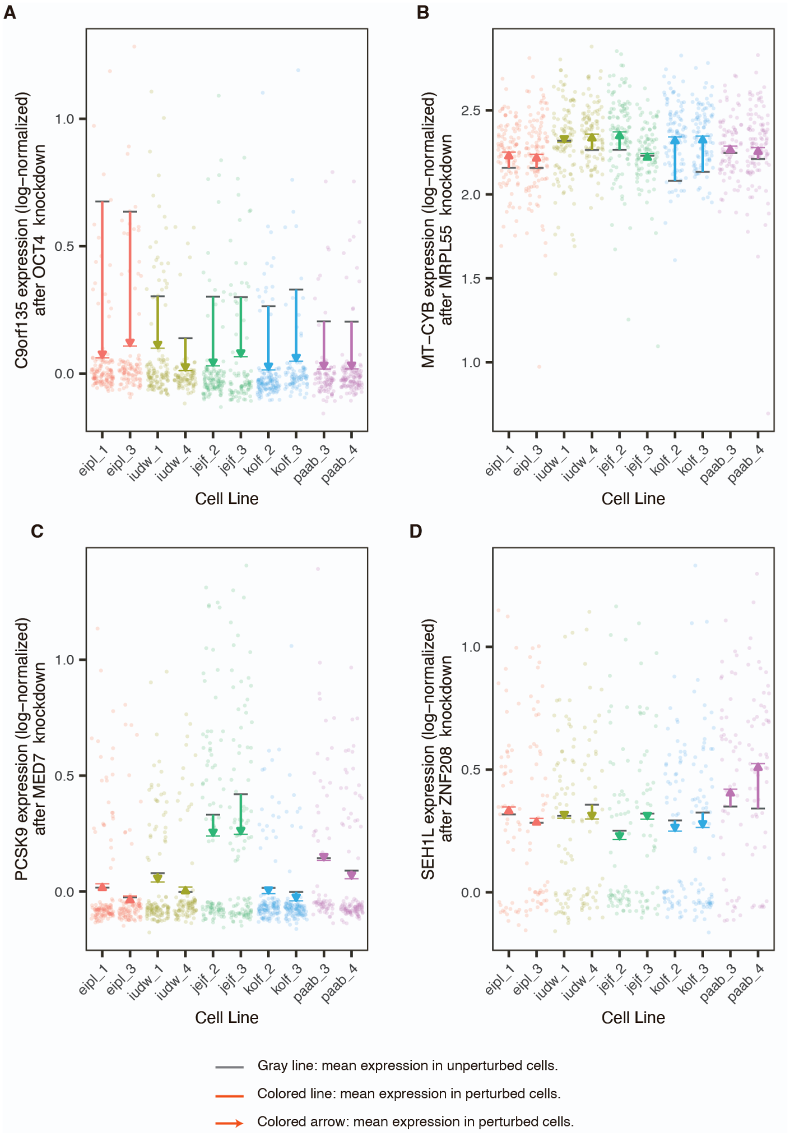

Figure S7 | Genetic background influences transcriptional response due to knockdown, related to Figure 3. A) An example of loss of heritability. *C9orf135* expression change due to knockdown of *OCT4*. C9orf135 expression (y-axis; log-normalized) in individual cells (markers) from different cell lines (x-axis, colors) with *OCT4* knockdown. Colored dash: mean expression in knockdown in cell line. Grey dash: mean expression in control cells in cell line. Colored arrow: median expression change in line in response to knockdown. B) As A), but expression change of *MRPL55* due to knockdown of *MRPL55*. C) Expression change of *PCSK9* due to knockdown of *MED7*. D) Expression change of *SEH1L* due to knockdown of the *trans* eQTL hotspot *ZNF208*.

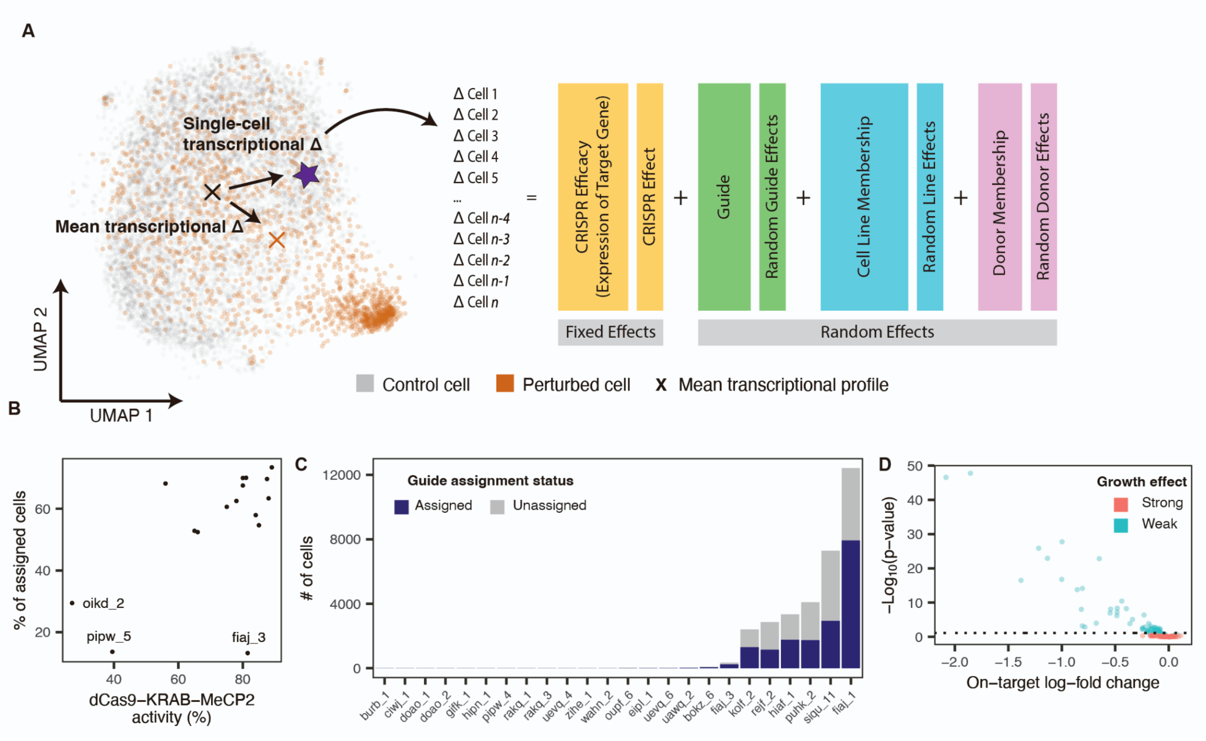

Figure S8 | Variation of transcriptional change due CRISPRi perturbation, related to Figure 4. A) Strategy for quantifying sources of variation in transcriptional response due to knockdown. B) dCas9-KRAB-MeCP2 activity vs. fraction of assigned cells. C) Number of cells recovered per cell line after 14 days of selection (y-axis) for different cell lines (x-axis) in a pilot experiment with early pooling of lines. D) Repression log-fold change (x-axis) and log-scale p-value (y-axis) of target gene (markers) in a pilot experiment with early pooling of lines and late sequencing time point (14 days post-infection).

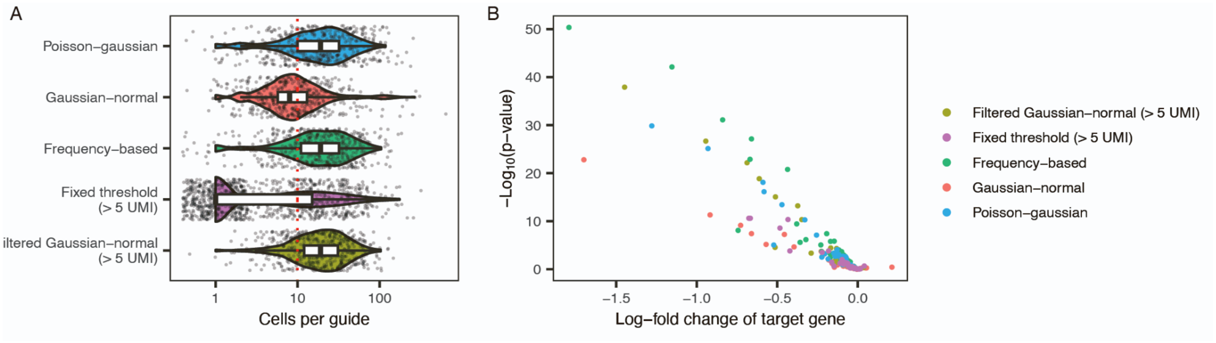

Figure S9 | Comparison of guide assignment strategies, related to STAR Methods. We utilized a Poisson-Gaussian model[22], a Gaussian-Gaussian model[81] adopted from previous work, as well as assigning cells to a guide if and only if more than a fixed threshold (> 5) of guide UMIs were detected (fixed threshold), if and only if the fraction of a guide compared to all UMIs in a given cell was greater than a given threshold (ratio) and a modified version of the Gaussian-Gaussian model where assignments were further filtered so that assignments based on fewer than 5 guide UMIs were disregarded (filtered Gaussian-Gaussian). To evaluate the quality of each guide assignment method, we considered A) the number of assigned cells per guide and B) on-target repression.

Supplementary tables

Primer ID	Sequence	Subpool
512	GAGCACAGATGGCACAAATG	GenomeWideScreen_Fitness_1; TargetedScreen
513	GAGCACAGATGGCAAACAGG	GenomeWideScreen_Fitness_2
514	GAGCACAGATGGCACTTTTG	GenomeWideScreen_Fitness_3
515	GAGCACAGATGGCTCTACGC	GenomeWideScreen_Fitness_4
516	GAGCACAGATGGAGGACAGC	GenomeWideScreen_Fitness_5
517	GAGCACAGATGGGTTGCTTG	GenomeWideScreen_Fitness_6
518	GAGCACAGATGGACCTCGAG	GenomeWideScreen_Fitness_7
519	GAGCACAGATGGCCTACGAG	GenomeWideScreen_Fitness_8
520	GAGCACAGATGGTCATGTCG	GenomeWideScreen_Fitness_9
521	GAGCACAGATGGCCTGGTAC	GenomeWideScreen_Fitness_10
522	GAGCACAGATGGACTGGTTG	GenomeWideScreen_Fitness_11
523	GAGCACAGATGGCGGTAAAG	GenomeWideScreen_Fitness_12
889	ACAGCTCAGACGtcctgaga	GenomeWideScreen_Fitness_1 TargetedScreen
890	ACAGCTCAGACGggcttacc	GenomeWideScreen_Fitness_2
891	ACAGCTCAGACGcaacggct	GenomeWideScreen_Fitness_3
892	ACAGCTCAGACGgacggctc	GenomeWideScreen_Fitness_4
893	ACAGCTCAGACGagccgtcg	GenomeWideScreen_Fitness_5
894	ACAGCTCAGACGcatagcgc	GenomeWideScreen_Fitness_6
895	ACAGCTCAGACGgaacatgc	GenomeWideScreen_Fitness_7
896	ACAGCTCAGACGccgtacgt	GenomeWideScreen_Fitness_8
897	ACAGCTCAGACGaacgcgtc	GenomeWideScreen_Fitness_9
898	ACAGCTCAGACGacgttcag	GenomeWideScreen_Fitness_10
899	ACAGCTCAGACGcctacgga	GenomeWideScreen_Fitness_11
900	ACAGCTCAGACGtgactgcc	GenomeWideScreen_Fitness_12
1009	agcagatctgcgaaatcggatccgc	NA
1010	TTCCTCTGCCCTCgccctcccacacataaccag	NA
1012	acaagttaacttatctagatccggtggatcccg	NA
1013	caccggatctagataagttaacttgtttattgcagctt	NA
1014	cgtgataacttattatatatatattttcttgttatagatatc	NA
1016	CATGCGGTGACGTGGAGGAGAATCCTGGCCCAATGGTG AGCAAGGGCGAGGC	NA

Table S10 | Primers used, related to STAR Methods.

Supplementary note

Supplementary methods

CRISPR sequences used

gRNA capture primer

AAGCAGTGGTATCAACGCAGAGTACCAAGTTGATAACGGACTAGCC

T2A Sequence

tgtgtgggagggcGAGGGCAGAGGAAGTCTCCTAACATGCGGTGACGTGGAGGAGAATCC

BFP
ATGAGCGAGCTGATTAAGGAGAACATGCACATGAAGCTGTACATGGAGGGCACCGTGGAC
AACCATCACTTCAAGTGCACATCCGAGGGCGAAGGCAAGCCCTACGAGGGCACCCAGACC
ATGAGAATCAAGGTGGTCGAGGGCGGCCCTCTCCCCTTCGCCTTCGACATCCTGGCTACT
AGCTTCCTCTACGGCAGCAAGACCTTCATCAACCACACCCAGGGCATCCCCGACTTCTTC
AAGCAGTCCTTCCCTGAGGGCTTCACATGGGAGAGAGTCACCACATACGAGGACGGGGGC
GTGCTGACCGCTACCCAGGACACCAGCCTCCAGGACGGCTGCCTCATCTACAACGTCAAG
ATCAGAGGGGTGAACTTCACATCCAACGGCCCTGTGATGCAGAAGAAAACACTCGGCTGG
GAGGCCTTCACCGAGACGCTGTACCCCGCTGACGGCGGCCTGGAAGGCAGAAACGACATG
GCCCTGAAGCTCGTGGGCGGGAGCCATCTGATCGCAAACATCAAGACCACATATAGATCC
AAGAAACCCGCTAAGAACCTCAAGATGCCTGGCGTCTACTATGTGGACTACAGACTGGAA
AGAATCAAGGAGGCCAACAACGAGACCTACGTCGAGCAGCACGAGGTGGCAGTGGCCAGA
TACTGCGACCTCCCTAGCAAACTGGGGCACAAGCTTAAT

BSD
ATGGCCAAGCCTTTGTCTCAAGAAGAATCCACCCTCATTGAAAGAGCAACGGCTACAATC
AACAGCATCCCCATCTCTGAAGACTACAGCGTCGCCAGCGCAGCTCTCTCTAGCGACGGC
CGCATCTTCACTGGTGTCAATGTATATCATTTTACTGGGGGACCTTGTGCAGAACTCGTG
GTGCTGGGCACTGCTGCTGCTGCGGCAGCTGGCAACCTGACTTGTATCGTCGCGATCGGA
AATGAGAACAGGGGCATCTTGAGCCCCTGCGGACGGTGCCGACAGGTGCTTCTCGATCTG
CATCCTGGGATCAAAGCCATAGTGAAGGACAGTGATGGACAGCCGACGGCAGTTGGGATT
CGTGAATTGCTGCCCTCTGGTTATGTGTGGGAGGGC

dCas9-KRAB-MeCP2
ATGGACAAGAAGTACTCCATTGGGCTCGCTATCGGCACAAACAGCGTCGGCTGGGCCGTC
ATTACGGACGAGTACAAGGTGCCGAGCAAAAAATTCAAAGTTCTGGGCAATACCGATCGC
CACAGCATAAAGAAGAACCTCATTGGCGCCCTCCTGTTCGACTCCGGGGAGACGGCCGAA
GCCACGCGGCTCAAAAGAACAGCACGGCGCAGATATACCCGCAGAAAGAATCGGATCTGC
TACCTGCAGGAGATCTTTAGTAATGAGATGGCTAAGGTGGATGACTCTTTCTTCCATAGG
CTGGAGGAGTCCTTTTTGGTGGAGGAGGATAAAAAGCACGAGCGCCACCCAATCTTTGGC
AATATCGTGGACGAGGTGGCGTACCATGAAAAGTACCCAACCATATATCATCTGAGGAAG
AAGCTTGTAGACAGTACTGATAAGGCTGACTTGCGGTTGATCTATCTCGCGCTGGCGCAT
ATGATCAAATTTCGGGGACACTTCCTCATCGAGGGGGACCTGAACCCAGACAACAGCGAT
GTCGACAAACTCTTTATCCAACTGGTTCAGACTTACAATCAGCTTTTCGAAGAGAACCCG
ATCAACGCATCCGGAGTTGACGCCAAAGCAATCCTGAGCGCTAGGCTGTCCAAATCCCGG
CGGCTCGAAAACCTCATCGCACAGCTCCCTGGGGAGAAGAAGAACGGCCTGTTTGGTAAT

CTTATCGCCCTGTCACTCGGGCTGACCCCCAACTTTAAATCTAACTTCGACCTGGCCGAA
GATGCCAAGCTTCAACTGAGCAAAGACACCTACGATGATGATCTCGACAATCTGCTGGCC
CAGATCGGCGACCAGTACGCAGACCTTTTTTTGGCGGCAAAGAACCTGTCAGACGCCATT
CTGCTGAGTGATATTCTGCGAGTGAACACGGAGATCACCAAAGCTCCGCTGAGCGCTAGT
ATGATCAAGCGCTATGATGAGCACCACCAAGACTTGACTTTGCTGAAGGCCCTTGTCAGA
CAGCAACTGCCTGAGAAGTACAAGGAAATTTTCTTCGATCAGTCTAAAAATGGCTACGCC
GGATACATTGACGGCGGAGCAAGCCAGGAGGAATTTTACAAATTTATTAAGCCCATCTTG
GAAAAAATGGACGGCACCGAGGAGCTGCTGGTAAAGCTTAACAGAGAAGATCTGTTGCGC
AAACAGCGCACTTTCGACAATGGAAGCATCCCCCACCAGATTCACCTGGGCGAACTGCAC
GCTATCCTCAGGCGGCAAGAGGATTTCTACCCCTTTTTGAAAGATAACAGGGAAAAGATT
GAGAAAATCCTCACATTTCGGATACCCTACTATGTAGGCCCCCTCGCCCGGGGAAATTCC
AGATTCGCGTGGATGACTCGCAAATCAGAAGAGACCATCACTCCCTGGAACTTCGAGGAA
GTCGTGGATAAGGGGGCCTCTGCCCAGTCCTTCATCGAAAGGATGACTAACTTTGATAAA
AATCTGCCTAACGAAAAGGTGCTTCCTAAACACTCTCTGCTGTACGAGTACTTCACAGTT
TATAACGAGCTCACCAAGGTCAAATACGTCACAGAAGGGATGAGAAAGCCAGCATTCCTG
TCTGGAGAGCAGAAGAAAGCTATCGTGGACCTCCTCTTCAAGACGAACCGGAAAGTTACC
GTGAAACAGCTCAAAGAAGACTATTTCAAAAAGATTGAATGTTTCGACTCTGTTGAAATC
AGCGGAGTGGAGGATCGCTTCAACGCATCCCTGGGAACGTATCACGATCTCCTGAAAATC
ATTAAAGACAAGGACTTCCTGGACAATGAGGAGAACGAGGACATTCTTGAGGACATTGTC
CTCACCCTTACGTTGTTTGAAGATAGGGAGATGATTGAAGAACGCTTGAAAACTTACGCT
CATCTCTTCGACGACAAAGTCATGAAACAGCTCAAGAGGCGCCGATATACAGGATGGGGG
CGGCTGTCAAGAAAACTGATCAATGGGATCCGAGACAAGCAGAGTGGAAAGACAATCCTG
GATTTTCTTAAGTCCGATGGATTTGCCAACCGGAACTTCATGCAGTTGATCCATGATGAC
TCTCTCACCTTTAAGGAGGACATCCAGAAAGCACAAGTTTCTGGCCAGGGGGACAGTCTT
CACGAGCACATCGCTAATCTTGCAGGTAGCCCAGCTATCAAAAAGGGAATACTGCAGACC
GTTAAGGTCGTGGATGAACTCGTCAAAGTAATGGGAAGGCATAAGCCCGAGAATATCGTT
ATCGAGATGGCCCGAGAGAACCAAACTACCCAGAAGGGACAGAAGAACAGTAGGGAAAGG
ATGAAGAGGATTGAAGAGGGTATAAAAGAACTGGGGTCCCAAATCCTTAAGGAACACCCA
GTTGAAAACACCCAGCTTCAGAATGAGAAGCTCTACCTGTACTACCTGCAGAACGGCAGG
GACATGTACGTGGATCAGGAACTGGACATCAATCGGCTCTCCGACTACGACGTGGCTGCT
ATCGTGCCCCAGTCTTTTCTCAAAGATGATTCTATTGATAATAAAGTGTTGACAAGATCC
GATAAAGCTAGAGGGAAGAGTGATAACGTCCCCTCAGAAGAAGTTGTCAAGAAAATGAAA
AATTATTGGCGGCAGCTGCTGAACGCCAAACTGATCACACAACGGAAGTTCGATAATCTG
ACTAAGGCTGAACGAGGTGGCCTGTCTGAGTTGGATAAAGCCGGCTTCATCAAAAGGCAG
CTTGTTGAGACACGCCAGATCACCAAGCACGTGGCCCAAATTCTCGATTCACGCATGAAC
ACCAAGTACGATGAAAATGACAAACTGATTCGAGAGGTGAAAGTTATTACTCTGAAGTCT
AAGCTGGTCTCAGATTTCAGAAAGGACTTTCAGTTTTATAAGGTGAGAGAGATCAACAAT
TACCACCATGCGCATGATGCCTACCTGAATGCAGTGGTAGGCACTGCACTTATCAAAAAA
TATCCCAAGCTTGAATCTGAATTTGTTTACGGAGACTATAAAGTGTACGATGTTAGGAAA
ATGATCGCAAAGTCTGAGCAGGAAATAGGCAAGGCCACCGCTAAGTACTTCTTTTACAGC
AATATTATGAATTTTTTCAAGACCGAGATTACACTGGCCAATGGAGAGATTCGGAAGCGA
CCACTTATCGAAACAAACGGAGAAACAGGAGAAATCGTGTGGGACAAGGGTAGGGATTTC
GCGACAGTCCGGAAGGTCCTGTCCATGCCGCAGGTGAACATCGTTAAAAAGACCGAAGTA
CAGACCGGAGGCTTCTCCAAGGAAAGTATCCTCCCGAAAAGGAACAGCGACAAGCTGATC
GCACGCAAAAAAGATTGGGACCCCAAGAAATACGGCGGATTCGATTCTCCTACAGTCGCT
TACAGTGTACTGGTTGTGGCCAAAGTGGAGAAAGGGAAGTCTAAAAAACTCAAAAGCGTC
AAGGAACTGCTGGGCATCACAATCATGGAGCGATCAAGCTTCGAAAAAAACCCCATCGAC
TTTCTCGAGGCGAAAGGATATAAAGAGGTCAAAAAAGACCTCATCATTAAGCTTCCCAAG
TACTCTCTCTTTGAGCTTGAAAACGGCCGGAAACGAATGCTCGCTAGTGCGGGCGAGCTG
CAGAAAGGTAACGAGCTGGCACTGCCCTCTAAATACGTTAATTTCTTGTATCTGGCCAGC
CACTATGAAAAGCTCAAAGGGTCTCCCGAAGATAATGAGCAGAAGCAGCTGTTCGTGGAA
CAACACAAACACTACCTTGATGAGATCATCGAGCAAATAAGCGAATTCTCCAAAAGAGTG

ATCCTCGCCGACGCTAACCTCGATAAGGTGCTTTCTGCTTACAATAAGCACAGGGATAAG
CCCATCAGGGAGCAGGCAGAAAACATTATCCACTTGTTTACTCTGACCAACTTGGGCGCG
CCTGCAGCCTTCAAGTACTTCGACACCACCATAGACAGAAAGCGGTACACCTCTACAAAG
GAGGTCCTGGACGCCACACTGATTCATCAGTCAATTACGGGGCTCTATGAAACAAGAATC
GACCTCTCTCAGCTCGGTGGAGAC

mScarlet
ATGCGGTTCAAGGTGCACATGGAGGGCTCCATGAACGGCCACGAGTTCGAGATCGAGGGC
GAGGGCGAGGGCCGCCCCTACGAGGGCACCCAGACCGCCAAGCTGAAGGTGACCAAGGGT
GGCCCCCTGCCCTTCTCCTGGGACATCCTGTCCCCTCAGTTCATGTACGGCTCCAGGGCC
TTCACCAAGCACCCCGCCGACATCCCCGACTACTATAAGCAGTCCTTCCCCGAGGGCTTC
AAGTGGGAGCGCGTGATGAACTTCGAGGACGGCGGCGCCGTGACCGTGACCCAGGACACC
TCCCTGGAGGACGGCACCCTGATCTACAAGGTGAAGCTCCGCGGCACCAACTTCCCTCCT
GACGGCCCCGTAATGCAGAAGAAGACAATGGGCTGGGAAGCGTCCACCGAGCGGTTGTAC
CCCGAGGACGGCGTGCTGAAGGGCGACATTAAGATGGCCCTGCGCCTGAAGGACGGCGGC
CGCTACCTGGCGGACTTCAAGACCACCTACAAGGCCAAGAAGCCCGTGCAGATGCCCGGC
GCCTACAACGTCGACCGCAAGTTGGACATCACCTCCCACAACGAGGACTACACCGTGGTG
GAACAGTACGAACGCTCCGAGGGCCGCCACTCCACCGGCGGCATGGACGAGCTGTACAAG
TCCGGACTCAGATCTCGAGCTCAAGCTTCGAATTCTGCAGTCGACGGTACCGCGGGCCCG
GGATCCACCGGATCTAGA

PURO
ATGACCGAGTACAAGCCCACGGTGCGCCTCGCCACCCGCGACGACGTCCCCAGGGCCGTA
CGCACCCTCGCCGCCGCGTTCGCCGACTACCCCGCCACGCGCCACACCGTCGATCCGGAC
CGCCACATCGAGCGGGTCACCGAGCTGCAAGAACTCTTCCTCACGCGCGTCGGGCTCGAC
ATCGGCAAGGTGTGGGTCGCGGACGACGGCGCCGCGGTGGCGGTCTGGACCACGCCGGAG
AGCGTCGAAGCGGGGGCGGTGTTCGCCGAGATCGGCCCGCGCATGGCCGAGTTGAGCGGT
TCCCGGCTGGCCGCGCAGCAACAGATGGAAGGCCTCCTGGCGCCGCACCGGCCCAAGGAG
CCCGCGTGGTTCCTGGCCACCGTCGGCGTCTCGCCCGACCACCAGGGCAAGGGTCTGGGC
AGCGCCGTCGTGCTCCCCGGAGTGGAGGCGGCCGAGCGCGCCGGGGTGCCCGCCTTCCTG
GAGACCTCCGCGCCCCGCAACCTCCCCTTCTACGAGCGGCTCGGCTTCACCGTCACCGCC
GACGTCGAGGTGCCCGAAGGACCGCGCACCTGGTGCATGACCCGCAAGCCCGGTGCC
